# Low-Level Mouse DNA in Conditioned Medium Generates False Positive Cross-Species Contamination Results in Human Organoid Cultures

**DOI:** 10.3389/fcell.2020.587107

**Published:** 2020-11-06

**Authors:** Margaret S. Bohm, Michael K. Dame, Joseph Boyd, Kevin Su, Angeline Wu, Durga Attili, Vi Chu, Justin A. Colacino, Jason R. Spence

**Affiliations:** ^1^Division of Gastroenterology, Department of Internal Medicine, The University of Michigan Medical School, Ann Arbor, MI, United States; ^2^Millipore-Sigma Corporation, Temecula, CA, United States; ^3^Department of Cell and Developmental Biology, The University of Michigan Medical School, Ann Arbor, MI, United States; ^4^Department of Environmental Health Sciences, The University of Michigan School of Public Health, Ann Arbor, MI, United States; ^5^Department of Nutritional Sciences, The University of Michigan School of Public Health, Ann Arbor, MI, United States; ^6^Center for Computational Medicine and Bioinformatics, The University of Michigan Medical School, Ann Arbor, MI, United States; ^7^Department of Biomedical Engineering, The University of Michigan Medical School, Ann Arbor, MI, United States

**Keywords:** cell-free DNA, cfDNA, colonoid, conditioned medium, enteroid, murine contamination, organoid, cell line authentication

## Abstract

Cell line authentication is critical for preventing the use of mixed or misidentified cell lines in research. Current efforts include short tandem repeat (STR) analysis and PCR-based assays to detect mixed species cultures. Using PCR analysis with mouse-specific primers, we identified contaminating mouse DNA in growth factor conditioned medium (CM) derived from the L-WRN cell line (L-WRN CM), as well as in human organoid cultures maintained in the L-WRN CM. DNA isolated from L-WRN CM matched the L-WRN cell signature by STR analysis. Organoid lines that were positive for murine DNA by PCR were further analyzed via bulk RNA-sequencing and transcripts were aligned to the human and mouse genomes. RNA analysis failed to detect mouse-specific gene expression above background levels, suggesting no viable murine cells were present in the organoid cultures. We interpret our data to show conclusive evidence that mouse cell-derived CM can be a source of contaminating murine DNA detected in human organoid cultures, even though live, transcriptionally-active murine cells are not present. Together, our findings suggest that multiple methods may be required to authenticate human organoid or cell lines and urges cautious interpretation of DNA-based PCR cell line authentication results.

## Introduction

As the use of primary human 3-dimensional (3D) organoids in research increases, so does the need for stringent cell line authentication measures for these cultures. The use of misidentified or contaminated cell lines has been a concern for more than 70 years and has called into question experimental conclusions, lead to poor reproducibility, wasted research dollars, and led to manuscript retractions (American Type Culture Collection Standards Development Organization Workgroup ASN-0002, 2010). To address these issues, guidelines have been submitted by many organizations to ensure proper cell identification (Geraghty et al., 2014). However, despite the development of guidelines that suggest regular testing (Barallon et al., 2010), many contaminated lines continue to be used for *in vitro* research. Nardone (2007) proposed that publishers and grant agencies effect change through publishing and funding policy, and gradually this has taken hold (NIH, 2015). Testing for cell line contamination has become a priority for researchers, and efforts have centered around detecting mixed or misidentified cell lines (Babic et al., 2019).

The current principle method of cell line authentication is routine short tandem repeat (STR) analysis of nuclear DNA, which is a vital tool for tracing the origins of a cell line back to a single source. Previous reports of cellular contamination have overwhelmingly been the result of intra-species contamination, such as interloping HeLa cells, which are easily recognized by STR analysis (Capes-Davis et al., 2010). However, STR analysis relies on the comparison of the test culture profile to a known profile of the source culture. If there is no source profile, STR analysis can only identify contamination of other known cell line profiles such as HeLa cells (Schweppe et al., 2008). Also, STR profiling fails to detect cellular contamination from other species (American Type Culture Collection Standards Development Organization Workgroup ASN-0002, 2010) or clonal drift from cultures contaminated many years prior (Schweppe et al., 2008). To address the limitations of STR analysis in detecting mixed species cultures, PCR-based assays can be a tool used to amplify species-specific genes.

Cell line authenticity is a significant concern in research utilizing human-derived, 3D epithelium-only organoids which can be derived from many organ systems. These lines, established from surgically resected tissue or biopsies, require highly specialized medium and other culture components for *in vitro* self-renewal. The requisite growth factors can be cost-prohibitive when purchased commercially, and this has driven the use of media conditioned with growth factors by feeder cells. For example, ligands from the WNT family of proteins are required to support the stem cell niche of gastrointestinal epithelial cells (Kaushik et al., 2018), but these ligands lose activity when introduced as purified recombinant proteins (Mihara et al., 2016). Therefore, WNT-conditioned medium is a common way to provide these vital growth factors. Conditioned medium (CM) is produced by recombinant murine cell lines (Jung et al., 2011; Miyoshi et al., 2012; Tsai et al., 2018), which increases chances for cell contamination. Numerous other culture components also have the potential to introduce contamination, such as the use of murine 3T3-J2 fibroblast feeder layers [breast, prostate, lung, cervix, oral, ovary, skin, salivary gland, kidney, thyroid (Liu et al., 2017), and Barrett’s esophagus (Yamamoto et al., 2016)] and Matrigel^TM^ extracellular matrix (Sato and Clevers, 2015). Widely used in gastrointestinal organoid research, mouse L-cells (Willert et al., 2003) have been modified into “L-WRN” cells, producing high levels of WNT3A, R-SPONDIN3, and NOGGIN (Miyoshi et al., 2012). Here, we demonstrate that low-level cell-free DNA (cfDNA) found in murine-produced CM from L-WRN cells can give false-positive contamination results in PCR-based analysis of organoid cultures.

## Methods

### Establishment and Maintenance of Human Enteroid Cultures

Enteroid cultures were established and maintained as previously described (Jung et al., 2011; Dame et al., 2018). Briefly, normal gastrointestinal tissues were collected from deceased donors through the Gift of Life, Michigan with institutional IRB approval, and the mucosa was separated from resected surgical tissue and incubated with 10 mM dithiothreitol for 15 min (Sigma-Aldrich) prior to cold incubation with 8 mM ethylenediaminetetraacetic acid (Sigma-Aldrich) for 75 min. Pure crypts were detached from the *lamina propria* by snap-shaking, washed three times in cold DPBS and cryopreserved in growth medium containing 10% FBS and 10% DMSO. Cryopreserved crypts were later seeded into 8 mg/mL Matrigel (Corning) and expanded as described previously (Tsai et al., 2018).

L-WRN conditioned medium was produced as previously described in detail (Tsai et al., 2018). Enteroid cultures were established and maintained in 50% L-WRN conditioned medium [produced by L-WRN cells to contain WNT3A, RSPONDIN3, and NOGGIN and including 20% FBS (final 10%) as described in Miyoshi and Stappenbeck (2013)] and 50% basal medium composed of advanced Dulbecco’s modified Eagle Medium/F12, with final concentrations of 2 mM GlutaMAX (Invitrogen), 10 mM HEPES (Invitrogen), 1x N2 supplement (Invitrogen), 1x B27 supplement without retinyl acetate (Invitrogen), 100 ng/mL EGF (R&D), 1 mM N-Acetyl-L-Cysteine, 50 units/mL penicillin/streptomycin (Invitrogen), 100 μg/mL Primocin (InvivoGen), the rho kinase inhibitor Y27632, 10 μM (Tocris), the TGF-β inhibitor A83-01, 500 nM (Tocris), and the p38 inhibitor SB202190, 10 μM (Tocris). Cultures were supplemented with the GSK-3 inhibitor CHIR99021, 2.5 μM (Tocris) for the first 10 days of establishment. Nicotinamide, 10 mM, was used at the initial establishment of the specimens and then removed for continued expansion of the lines (Bartfeld et al., 2015). Medium changes occurred daily. Cultures were split every 5–7 days using cold mechanical dissociation from previous Matrigel by a 1 mL pipet, followed by re-plating in fresh Matrigel in 10 μL droplets. CHIR99021, 2.5 μM, was included for 24 h following each passage. All lines were confirmed to be negative for mycoplasma and were tested for the presence of viruses (CLEAR with Infectious Disease PCR Panel, Charles River Laboratories).

### DNA Isolation and RNA Sequencing

DNA isolation was conducted using Wizard Genomic DNA Purification kit (A1120, Promega) following mechanical dissociation of enteroids from Matrigel via trituration. RNA isolation was conducted using the RNeasy Micro Kit (84004, Qiagen) with on-column DNase digestion after mechanical dissociation of enteroids from Matrigel via trituration and a 15 min 4°C incubation in 2 mM EDTA with three subsequent washes in cold DPBS. RNA concentration and quality were determined using a Nanodrop (Thermo Fisher Scientific) and Bioanalyzer (Agilent), and prepared for library generation with Takara SMARTer Stranded Total RNA Sample Prep Kit (634876, Takara Bio USA). RNA sequences were generated for 50-bp single-end reads across 10 lanes on an Illumina HiSeq 2500 by the University of Michigan DNA Sequencing Core. Proportion of transcripts derived from mouse samples were analyzed based on the method developed by Callari et al. (2018). Sequencing reads were aligned to a combined mouse *mm10* and human *hg19* reference genome (Macosko et al., 2015) using STAR (Dobin et al., 2013). Aligned reads were simultaneously assigned to the human or mouse transcriptome using featureCounts (Liao et al., 2014), and the proportion of reads aligning uniquely to human or mouse genes were calculated for each sample. Control samples of uncultured human mammary gland and conditionally reprogrammed human mammary cells grown on a mouse 3T3-J2 feeder layer were established and processed as described in Thong et al. (2020). The raw sequence data is publicly available at ArrayExpress archive under accession number E-MTAB-9339.^1^

### STR Analysis and PCR Detection of Mouse Genes

Mouse STR analysis was completed by ATCC according to their Mouse Cell Authentication Service (137-XV, ATCC). Human STR analysis was carried out at the University of Michigan DNA Sequencing Core with the AMPFLSTR Identifier Plus Assay (Applied Biosystems) to identify human genomic DNA for 15 tetranucleotide repeat loci and the amelogenin gender determination marker run on the 3730XL Genetic Analyzer (Applied Biosystems). Commercial PCR analysis was performed by Charles River Laboratories according to their Cell Line Examination and Report with Infectious Disease PCR Panel (CLEAR PCR Panel, Charles River Laboratories). PCR analysis of conditioned medium for mouse-specific *V1rh10* was conducted utilizing primers specified by Holder and Cooper (2011) (Forward: TTCAGGGTGCTATGGGAGGGGC Reverse: GCCCATCCCTGTGAATCAGCACA, 300 bp product). Primers were produced by IDT technologies, and 32 cycles of 10 ng DNA reactions were completed at 60°C annealing temperature on C1000 Touch Thermal Cycler (M0488S, Bio-Rad) using One*Taq* Hot Start Quick-Load PCR kit (New England BioLabs). Results were analyzed by gel electrophoresis on a 1.5% agarose gel that was visualized on Alphaimager 2200 (Alpha Innotech). Validation of conditioned medium analysis and investigation of commercial murine-derived culture components (Matrigel^TM^, IntestiCult-human^TM^) was conducted via PCR amplification of the mouse-specific *Ptger2* gene (Alcoser et al., 2011) (Forward: CCTGCTGCTTATCGTGGCTG, Reverse: GCCAGGAGAATGAGGTGGTC, 189 bp product). *Ptger2* primers were produced by Sigma-Aldrich, and 40 cycles were completed at 60°C annealing temperature on MyCycler Thermal Cycler (Bio-Rad) using Q5 DNA Polymerase PCR kit (New England BioLabs). Gel electrophoresis was carried out on a 1% agarose gel that was visualized on ChemiDoc MP imager (Bio-Rad).

## Results

We conducted STR and PCR analyses during routine authentication of samples from a human organoid biobank in accordance with accepted cell line authentication practices (Geraghty et al., 2014; Dame et al., 2018). STR profiles indicated that each of the lines matched their source tissue, maintaining their unique identities with no cross-contamination detected between lines (Supplementary Table S1). However, in a commercially available PCR-based assay, murine DNA was amplified by species-specific primers and demonstrated that all human organoid lines analyzed (*n* = 14) were positive for mouse contamination (Table 1). This same assay did not detect mouse contamination in a primary human colon tissue sample that was not cultured, confirming the species-specificity of the PCR-based test (Table 1).

**TABLE 1:**
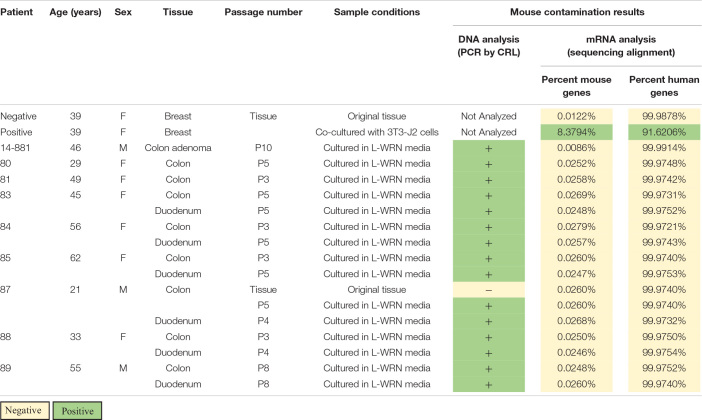
Genetic analysis of human organoid lines for evidence of active murine contamination; DNA analysis [Charles River Laboratories (CRL) CLEAR panel] has a limit of detection of 0.5% contamination while mRNA analysis has a contamination threshold of 1.2% mouse genes.

**FIGURE 1:**
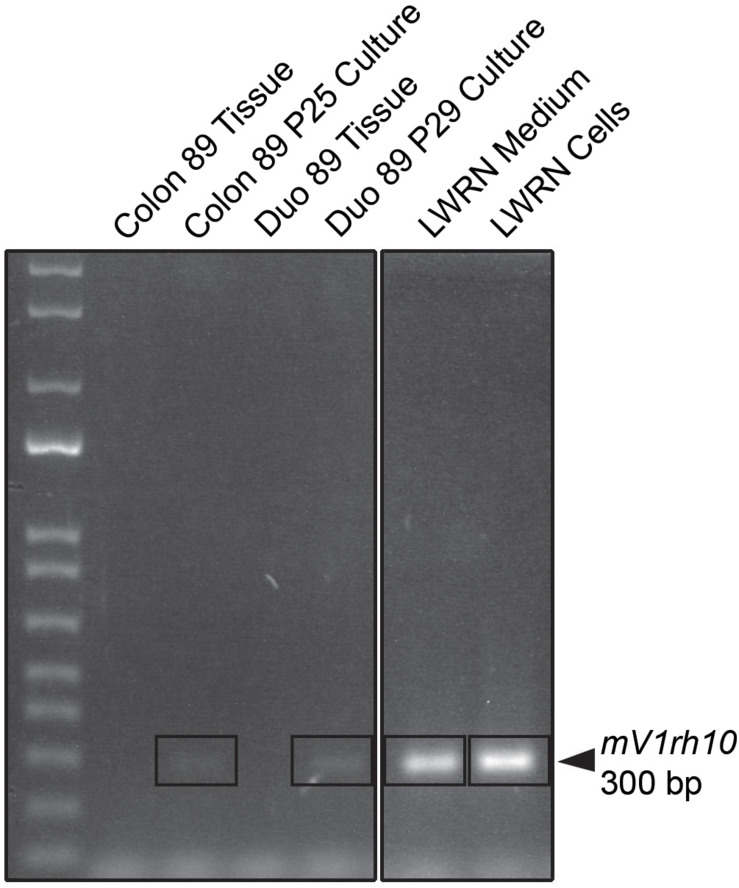
PCR product analysis by agarose gel electrophoresis of L-WRN conditioned medium as a potential mouse contamination source. Murine DNA is identified with the mouse-specific marker *V1rh10* in the L-WRN conditioned medium and in the L-WRN CM-cultured organoid lines, Colon 89 and Duodenum 89. The patient tissue samples for both Colon and Duodenum 89 were negative for the marker. Patient 89 served as a representative sample of all lines analyzed via PCR for murine contamination, and the mouse L-WRN cells serve as a positive control for this analysis (samples were run on the same gel).

To identify potential sources of mouse contamination, we tested an aliquot of CM for the presence of cfDNA by PCR and STR profiling. To carry out this analysis, we utilized a fresh aliquot of CM that had never been used to culture enteroids. Following DNA isolation from the medium, we carried out PCR using mouse-specific primers for the *V1rh10* gene as previously described by others (Holder and Cooper, 2011) and detected murine DNA in the CM (Figure 1). To confirm the finding that murine DNA was detected in L-WRN-cultured organoids only, we identified *V1rh10* amplification in samples of cultured colonoids and enteroids; the matched primary tissue samples did not amplify this marker (Figure 1). We then generated a mouse STR profile from the CM to determine the source of this cfDNA (Table 2). We compared this profile to the STR profiles of the L-WRN cells used to make the CM and to the parent L-cells (made available by ATCC). The L-WRN cells themselves matched the profile for L-cells with greater than 80% similarity between identified alleles (Reid et al., 2004). The STR profile of the cfDNA from the L-WRN CM contained 8 loci with intact alleles out of 18 potential loci, of which 100% of the alleles identified in the CM matched the L-cell and L-WRN cell profiles.

**TABLE 2:**
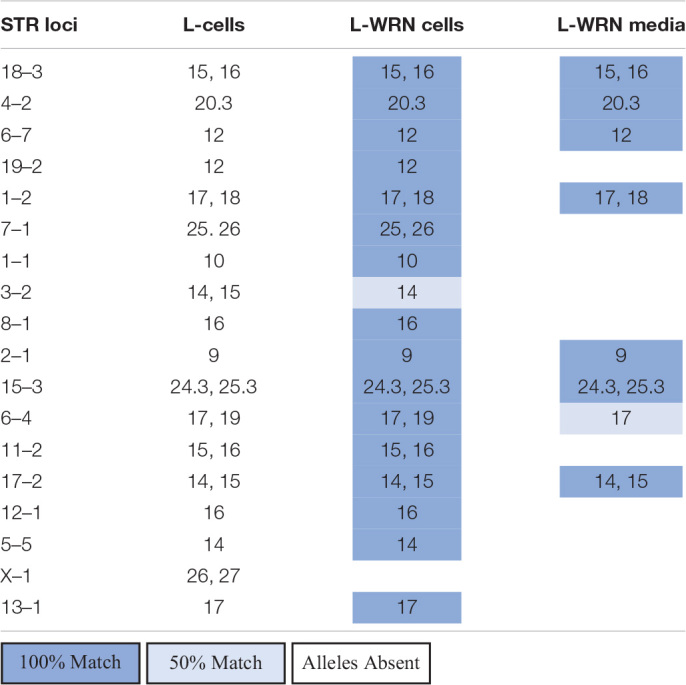
Comparison of free DNA found in L-WRN CM to L-WRN cells and the parent L-cells via STR analysis.

We interrogated the same human enteroid cultures for active murine RNA transcription using bulk RNA sequencing (RNA-seq). Sequenced reads were aligned to both the human and mouse genomes to determine the percentage of mouse transcripts relative to human (Table 1). Included in our analysis was a positive control sample of human mammary tissue co-cultured with mouse 3T3-J2 feeder cells. We observed that 8.38% of transcripts aligned to the mouse genome in the co-cultures, which is above the 1.2% threshold suggested as an indicator of actively transcribing cells (Callari et al., 2018). Conversely, a 0.012% mouse genome alignment was found in an uncultured sample of the same mammary tissue, setting the threshold level of background for reads that will errantly map to the mouse genome in a purely human sample. Organoid cultures showed aligned mouse reads similar to the background level found in controls (0.0086–0.028%, Table 1, *n* = 14). Human genome alignments for those same samples ranged from 99.97–99.99%. Collectively, these data show that active transcription in human organoid cultures comes from human but not mouse cells, providing strong evidence that murine cells are not present in these cultures whereas murine cfDNA from LWRN CM is present.

## Discussion

Following the detection of murine DNA in a series of human organoid lines (Table 1), we examined the components used to maintain our cultures to identify potential contamination sources. Since L-WRN CM is derived from mouse cells, we reasoned that the murine contamination may result from release of cellular contents during the conditioning process. This supposition was supported by the detection of murine DNA in a fresh aliquot of L-WRN CM (Figure 1) and further confirmed by STR profiling (Table 2). There were fewer intact alleles in the CM STR profile than the L-WRN cell profile, likely because cfDNA has shorter intact DNA fragments than nuclear DNA due to vulnerability to degrative enzymes (Diaz et al., 2016). However, 100% of the alleles identified in the CM matched both the L-cell and L-WRN cell profiles, strongly suggesting that the cfDNA in the CM originated from the L-WRN cells utilized in production. This finding suggested that the murine DNA found in our organoid cultures originated from cfDNA in the CM.

To rule out the possibility that live mouse cells exist in the enteroid cultures, we analyzed RNA sequencing data from each of the cell lines that had been positive for murine contamination. Following determination of the percentage of murine mRNA present in each culture, every culture was definitively negative for murine mRNA (Table 1). This data supports the conclusion there is no active murine mRNA transcription in organoid cultures. Coupled with the identification of free mouse DNA present in CM, our data suggests that the CM used to culture our organoid lines contained enough murine cfDNA to generate a false positive cross-species contamination result on our PCR-based assays. Multiple experimental assays, in this case PCR analysis, STR profiling, and bulk RNA-sequencing analysis, need to be performed in concert to delineate cell contamination from this cfDNA contamination.

Since murine L-WRN cells are used for CM production, it is not surprising that cellular material, including cfDNA, is found in the L-WRN CM itself. During preparation of CM, basal medium is added to confluent monolayers of murine cells, harvested, and refreshed daily for up to 12 days. In the standard course of L-WRN culture, murine cells continuously slough off into the medium. Any intact cells are discarded through sedimentation and filtration prior to medium storage, but low levels of intracellular contents released from fragmented cells can remain suspended in the medium. DNA in particular is stable at the temperatures achieved during CM storage (−80°C) and culture use (37°C), allowing fragments to remain intact for identification by PCR-based assays, as shown above. Murine DNA was also identified in commercially available organoid culture components via PCR analysis (Supplementary Figure S1). We interpret our data to show conclusive evidence that mouse cell-derived CM can contribute murine DNA at high enough concentrations that it is detectable in human organoid cultures. We have also shown that transcriptionally active murine cells are not present in those same organoid cultures. As a result, we determine that the murine contamination detected by DNA-based cross-species contamination testing is cfDNA introduced by the CM used to maintain the lines. Organoid culture of many different organs utilize murine-derived components and could be vulnerable to the same cfDNA contamination that we found in the L-WRN CM. Our findings suggest that multiple methods should be utilized to authenticate cell lines.

## Data Availability Statement

The datasets presented in this study can be found at https://www.ebi.ac.uk/arrayexpress/E-MTAB-9339.

## Ethics Statement

The studies involving human participants were reviewed and approved by the Institutional Review Boards of the University of Michigan Medical School (IRBMED). Tissues were collected from deceased donors through Gift of Life, Michigan. Written informed consent for participation was not required for this study in accordance with the national legislation and the institutional requirements.

## Author Contributions

MB, MD, VC, JC, and JS conceptualized the study. MB, JB, and DA conducted the experiments. MB, MD, JC, and JS wrote the first draft of the manuscript. MB, MD, DA, JB, JC, and JS revised the manuscript. All authors reviewed and approved the final version of the manuscript.

## Conflict of Interest

The authors acknowledge a commercial relationship with Millipore-Sigma Corporation. Millipore-Sigma Corporation did participate in this research as outlined in the author contributions. Findings by Millipore-Sigma were therein funded by Millipore-Sigma.

## Footnotes

1 https://www.ebi.ac.uk/arrayexpress/experiments/E-MTAB-9339/

## Supplementary Material

Supplementary Figure 1 PCR analysis of murine-derived commercial culture components for evidence of murine DNA. PCR analysis illustrated that murine DNA, indicated by the mouse-specific marker *Ptger2*, was present in both Matrigel^TM^ (Corning) and the organoid growth medium IntestiCult-human^TM^ (StemCell Technologies). *Ptger2* also confirmed the detectable presence of murine DNA in L-WRN conditioned medium by a second primer set.
