# Supplementary material for: Low-Level Mouse DNA in Conditioned Medium Generates False Positive Cross-Species Contamination Results in Human Organoid Cultures
Source: Front Cell Dev Biol. 2020 Nov 6;8:587107. doi: 10.3389/fcell.2020.587107 (PMC7677229; doi:10.3389/fcell.2020.587107)
Supplement: Supplementary Figure 1 — PCR analysis of murine-derived commercial culture components for evidence of murine DNA. PCR analysis illustrated that murine DNA, indicated by the mouse-specific marker Ptger2, was present in both MatrigelTM (Corning) and the organoid growth medium IntestiCult-humanTM (StemCell Technologies). Ptger2 also confirmed the detectable presence of murine DNA in L-WRN conditioned medium by a second primer set. [file Presentation_1.pptx]

## Slide 1
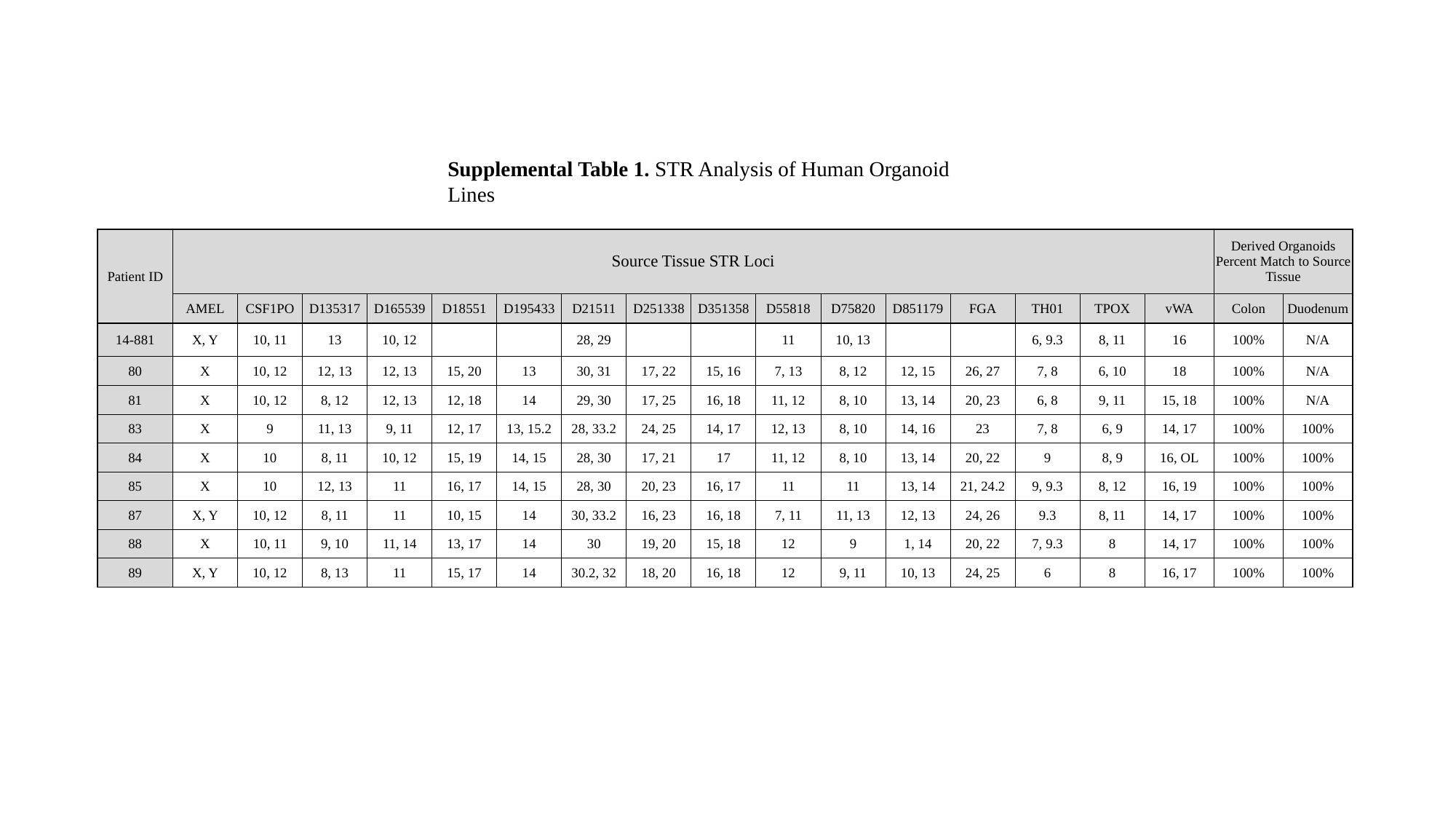

Supplemental Table 1. STR Analysis of Human Organoid Lines
| Patient ID | Source Tissue STR Loci | | | | | | | | | | | | | | | | Derived Organoids Percent Match to Source Tissue | |
| --- | --- | --- | --- | --- | --- | --- | --- | --- | --- | --- | --- | --- | --- | --- | --- | --- | --- | --- |
| | AMEL | CSF1PO | D135317 | D165539 | D18551 | D195433 | D21511 | D251338 | D351358 | D55818 | D75820 | D851179 | FGA | TH01 | TPOX | vWA | Colon | Duodenum |
| 14-881 | X, Y | 10, 11 | 13 | 10, 12 | | | 28, 29 | | | 11 | 10, 13 | | | 6, 9.3 | 8, 11 | 16 | 100% | N/A |
| 80 | X | 10, 12 | 12, 13 | 12, 13 | 15, 20 | 13 | 30, 31 | 17, 22 | 15, 16 | 7, 13 | 8, 12 | 12, 15 | 26, 27 | 7, 8 | 6, 10 | 18 | 100% | N/A |
| 81 | X | 10, 12 | 8, 12 | 12, 13 | 12, 18 | 14 | 29, 30 | 17, 25 | 16, 18 | 11, 12 | 8, 10 | 13, 14 | 20, 23 | 6, 8 | 9, 11 | 15, 18 | 100% | N/A |
| 83 | X | 9 | 11, 13 | 9, 11 | 12, 17 | 13, 15.2 | 28, 33.2 | 24, 25 | 14, 17 | 12, 13 | 8, 10 | 14, 16 | 23 | 7, 8 | 6, 9 | 14, 17 | 100% | 100% |
| 84 | X | 10 | 8, 11 | 10, 12 | 15, 19 | 14, 15 | 28, 30 | 17, 21 | 17 | 11, 12 | 8, 10 | 13, 14 | 20, 22 | 9 | 8, 9 | 16, OL | 100% | 100% |
| 85 | X | 10 | 12, 13 | 11 | 16, 17 | 14, 15 | 28, 30 | 20, 23 | 16, 17 | 11 | 11 | 13, 14 | 21, 24.2 | 9, 9.3 | 8, 12 | 16, 19 | 100% | 100% |
| 87 | X, Y | 10, 12 | 8, 11 | 11 | 10, 15 | 14 | 30, 33.2 | 16, 23 | 16, 18 | 7, 11 | 11, 13 | 12, 13 | 24, 26 | 9.3 | 8, 11 | 14, 17 | 100% | 100% |
| 88 | X | 10, 11 | 9, 10 | 11, 14 | 13, 17 | 14 | 30 | 19, 20 | 15, 18 | 12 | 9 | 1, 14 | 20, 22 | 7, 9.3 | 8 | 14, 17 | 100% | 100% |
| 89 | X, Y | 10, 12 | 8, 13 | 11 | 15, 17 | 14 | 30.2, 32 | 18, 20 | 16, 18 | 12 | 9, 11 | 10, 13 | 24, 25 | 6 | 8 | 16, 17 | 100% | 100% |

## Slide 2
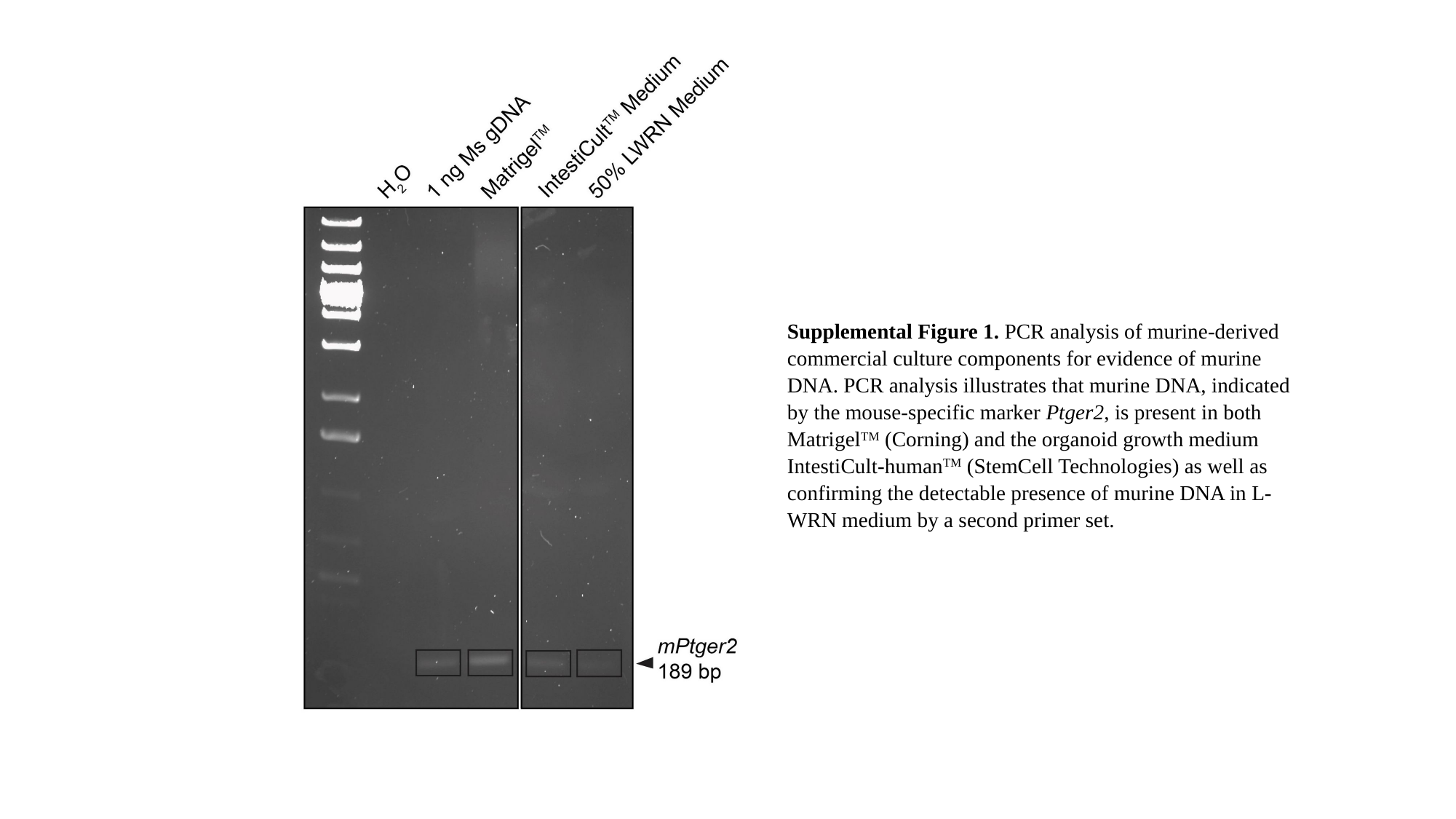

Supplemental Figure 1. PCR analysis of murine-derived commercial culture components for evidence of murine DNA. PCR analysis illustrates that murine DNA, indicated by the mouse-specific marker Ptger2, is present in both MatrigelTM (Corning) and the organoid growth medium IntestiCult-humanTM (StemCell Technologies) as well as confirming the detectable presence of murine DNA in L-WRN medium by a second primer set.
